# Alcohol Consumption and Ankle-to-Brachial Index: Results from the Cardiovascular Risk Survey

**DOI:** 10.1371/journal.pone.0015181

**Published:** 2010-12-02

**Authors:** Xiang Xie, Yi-Tong Ma, Yi-Ning Yang, Xiao-Mei Li, Fen Liu, Ding Huang, Zhen-Yan Fu, Xiang Ma, Bang-Dang Chen, Ying Huang

**Affiliations:** 1 Department of Cardiology, First Affiliated Hospital of Xinjiang Medical University, Urumqi, People's Republic of China; 2 Xinjiang Key Laboratory of Cardiovascular Disease Research, Urumqi, People's Republic of China; University of Padova, Italy

## Abstract

**Background and Methodology:**

A low ankle-to-brachial index (ABI) is a strong correlate of cardiovascular disease and subsequent mortality. The relationship between ABI and alcohol consumption remains unclear. Data are from the Cardiovascular Risk Survey (CRS), a multiple-ethnic, community-based, cross-sectional study of 14 618 Chinese people (5 757 Hans, 4 767 Uygurs, and 4 094 Kazakhs) aged 35 years and over at baseline from Oct. 2007 to March 2010. The relationship between alcohol intake and ABI was determined by use of analysis of covariance and multivariable regressions.

**Principal Findings:**

In men, alcohol consumption was significantly associated with ABI (*P*<0.001). After adjusted for the confounding factors, such as age, sex, ethnicity, body mass index, smoking, work stress, diabetes, and fasting blood glucose, the difference remained significant (*P*<0.001); either the unadjusted or multivariate-adjusted odds ratio (OR) for peripheral artery disease (PAD) was significantly higher in men who consumed >60.0 g/d [OR  = 3.857, (95% CI: 2.555–5.824); OR = 2.797, (95% CI: 1.106–3.129); OR = 2.878, (95% CI: 1.215–4.018); respectively] and was significantly lower in men who consumed 20.1–40.0 g/d [OR  = 0.330, (95% CI: 0.181–0.599); OR = 0.484, (95% CI: 0.065–0.894); OR = 0.478, (95% CI: 0.243–1.534); respectively] and 40.1–60.0 g/d [OR  = 0.306, (95% CI: 0.096–0.969); OR = 0.267, (95% CI: 0.087–0.886); OR = 0.203, (95% CI: 0.113–0.754); respectively] compared with never drinking, respectively (all *P*<0.01). Neither in unadjusted nor in multivariate-adjusted model was the association between ABI and alcohol consumption significant (all *P*>0.05) in women. Similarly, PAD was not correlated with alcohol intake in women (all *P*>0.05).

**Conclusions/Significance:**

Our results indicated that in Chinese men, alcohol consumption was associated with peripheral artery disease, and consumption of less than 60 g/d had an inverse association with peripheral atherosclerosis whereas consumption of 60 g/d or more had a positive association.

## Introduction

The ankle-brachial pressure index (ABI), which is the ratio of ankle to brachial systolic blood pressure, is the golden standard for the diagnosis of peripheral arterial disease (PAD) and is a highly specific method for the assessment of vascular risk in otherwise asymptomatic patients [1]. The American Heart Association (AHA) Prevention Conference V suggested that the ABI is a strong and independent risk factor for cardiovascular mortality and recommended it be used to detect subclinical disease in the prevention of cardiovascular mortality and stroke [2]–[3]. An ABI value <0.9 is widely acknowledged to indicate an abnormally low level [4] and several studies have shown that a low ABI was an independent predictor of increased risk of fatal myocardial infarction [5] and increased risk of cardiovascular disease (CVD) mortality [6]. And the ABI provides a simple measurement that can be performed in primary care settings without expensive or elaborate equipment or extensive training or experience [7]. Therefore, much research focus on the relationship between ABI and CVD recently [8], [9].

Many studies indicated that excessive alcohol intake maybe result in poor health outcome, especially cardiovascular disease [10], [11], but moderate drinking was associated with cardiovascular protective effects [12], [13]. Nevertheless, up to date, the relationship between ABI, an independent predictor of CVD, with alcohol intake remains unclear. In the present study, we investigated the relationship between alcohol consumption and ABI in Chinese population.

## Results

### Characteristics of Study Participants

The characteristics of study participants were shown in table 1. The mean age, BMI, triglyceride, total cholesterol, SBP, DBP and uric acid was difference between each ethnic group in both men and women (all *P*<0.01). The HDL cholesterol, was difference in men (*P* = 0.007) but not in women (*P* = 0.099), and fasting glucose was difference in men (*P*<0.001) but not in women (*P* = 0.113).

According to the amount of alcohol intake, we categorized the study population into six categories according to previous reported [14], whose characteristics were shown in table 2 and table 3. In both men and women, there were significantly different between each category in smoking, diabetes, hypertension, age, BMI, TG, TC, HDL-C (all *P*<0.05); There were no difference between each category in DBP, HDL-C, and LDL-C in men; There were no significant difference between each category in glucose, TC, HDL-C, LDL-C and DBP in women.

**Table 1:** Characteristics of participants.

	Men				Women			
	Han (2 471)	Uygur (1 678)	Kazakh (1 728)	*P* value	Han (2 686)	Uygur (2 330)	Kazakh (1 897)	*P* value
Smoking (n, %)	1546 (62.6)	730 (43.5)	965 (55.8)	<0.001	66 (2.46)	19 (0.82)	296 (15.6)	<0.001
Diabetes (n, %)	200 (8.09)	96 (5.72)	80 (4.63)	<0.001	158 (5.88)	120 (5.15)	43 (2.67)	<0.001
Hypertension (n, %)	1109 (44.88)	613 (36.53)	953 (55.15)	<0.001	1089 (40.54)	847 (36.35)	901 (47.50)	<0.001
Age (years)	51.2±12.8	52.2±13.4	48.7±11.9	<0.001	52.1±11.8	48.8±12.4	47.9±11.2	<0.001
BMI (kg/m^2^)	25.68±3.23	25.47±4.07	26.96±4.29	<0.001	24.50±3.60	25.97±4.51	26.23±5.19	<0.001
SBP (mmHg)	133.6±18.4	131.1±19.5	135.1±20.6	<0.001	130.3±20.6	130.5±21.9	138.2±25.9	0.009
DBP (mmHg)	87.3±15.1	80.4±14.6	89.9±18.4	<0.001	82.7±15.7	79.5±15.2	86.1±20.0	0.005
Uric acid (µmol/L)	341.7±86.9	284.7±77.2	294.7±77.3	<0.001	270.9±70.4	222.0±62.1	226.5±64.0	<0.001
Glucose (mmol/L)	5.44±1.96	4.93±1.86	5.23±1.61	<0.001	5.16±1.50	4.90±1.48	5.05±1.47	0.113
Triglyceride (mmol/L)	1.97±1.69	1.66±1.46	1.34±1.08	<0.001	1.46±1.10	1.60±1.14	1.09±0.65	<0.001
TC (mmol/L)	4.68±1.07	4.30±1.06	4.82±1.06	<0.001	4.70±1.09	4.40±1.13	4.73±1.13	<0.001
HDL –C (mmol/L)	1.24±0.45	1.26±0.50	1.28±0.44	0.007	1.27±0.47	1.26±0.45	1.29±0.45	0.099
LDL-C (mmol/L)	2.85±0.89	2.87±0.91	2.88±0.90	0.534	2.88±0.93	2.86±0.93	2.91±0.94	0.215

(Note:1 mm Hg  = 0.133 kPa; BMI, body mass index; SBP, systolic blood pressure; DBP, diastolic blood pressure; TC, total cholesterol; HDL-C, high density lipoproteins cholesterol; LDL-C, low density lipoproteins cholesterol).

**Table 2:** Cardiovascular Risks Factors According to Daily Alcohol Consumption Categories in Men.

	Categories of Alcohol consumption (g/day)						*P* value
	Never	Former	0.1–20.0	20.1–40.0	40.1–60	>60.0	
NO. of subjects	2791	420	1 440	795	214	217	
Smoking (n, %)	1782 (63.8)	272 (64.8)	513 (35.6)	148 (18.6)	113 (52.8)	103 (47.5)	<0.001
Diabetes (n, %)	243 (8.7)	30 (7.1)	142 (9.9)	70 (8.8)	29 (13.6)	32 (14.7)	0.411
Hypertension (n, %)	1773 (63.5)	221 (52.6)	382 (26.5)	176 (22.1)	77 (36.0)	146 (67.3)	<0.001
Age(years)	51.9±13.3	53.6±12.0	45.9±10.2	45.9±10.2	47.2±10.9	46.8±10.3	<0.001
BMI(kg/m^2^)	25.7±3.9	26.5±3.8	26.4±3.7	26.3±3.8	26.5±3.3	27.0±3.8	<0.001
SBP(mmHg)	134.9±21.2	136.0±20.0	133.9±19.1	132.6±18.7	136.5±18.6	137.2±18.7	0.048
DBP(mmHg)	85.4±16.7	85.6±15.5	86.9±15.9	88.3±15.0	88.1±16.0	89.9±15.8	<0.001
Glucose (mmol/L)	5.19±1.86	5.20±1.75	5.36±1.88	5.22±1.22	5.40±2.09	5.37±1.78	<0.001
TG (mmol/L)	1.55±1.34	1.83±1.50	1.95±1.55	2.20±1.50	2.11±2.98	2.17±1.77	<0.001
TC (mmol/L)	4.53±1.12	4.76±1.12	4.75±1.00	4.84±0.99	4.73±1.10	4.82±1.23	<0.001
HDL -C(mmol/L)	1.26±0.46	1.24±0.45	1.22±0.41	1.30±0.52	1.23±0.45	1.27±0.47	0.243
LDL-C (mmol/L)	2.86±0.90	2.92±0.95	2.84±0.89	2.83±0.86	2.78±0.83	2.93±0.89	0.311

Note:1 mm Hg  = 0.133 kPa; BMI, body mass index; SBP, systolic blood pressure; DBP, diastolic blood pressure; LDL, low-density lipoprotein; HDL, high-density lipoprotein; TG, Triglyceride; TC, Total cholesterol.

**Table 3:** Cardiovascular Risks Factors According to Daily Alcohol Consumption Categories in Women.

	Categories of Alcohol consumption (g/day)						*P* value
	Never	Former	0.1–5.0	5.1–10.0	10.1–20	>20.0	
NO. of subjects	5 147	82	1038	174	271	201	
Smoking (n, %)	355 (6.9)	26 (31.7)	270 (26.0)	45 (25.9)	51 (18.8)	64 (31.8)	<0.001
Diabetes (n, %)	316 (6.1)	8 (9.8)	57 (5.5)	21 (7.1)	28 (13.9)	19 (9.5)	<0.001
Hypertension (n, %)	2806 (54.5)	27 (32.9)	404 (38.9)	76 (43.7)	121 (44.6)	87 (43.3)	<0.001
Age (years)	49.8±12.0	43.4±12.4	46.6±10.6	48.3±10.3	49.6±8.2	46.4±8.2	0.036
BMI (kg/m^2^)	25.5±4.5	24.0±3.1	23.6±3.0	24.7±2.0	22.4±1.7	25.8±3.0	0.045
SBP (mmHg)	132.6±22.9	125.4±20.4	123.6±20.0	129.0±16.7	132.7±21.1	139.5±19.9	0.013
DBP (mmHg)	82.5±17.0	81.4±15.9	79.7±18.5	82.4±12.4	77.0±13.9	91.3±24.1	0.164
Glucose (mmol/L)	5.04±1.49	4.96±1.24	5.37±2.19	4.39±0.33	4.53±0.39	4.97±1.14	0.629
TG (mmol/L)	1.40±1.03	1.48±1.19	1.51±0.98	1.70±1.23	1.35±1.15	2.22±1.50	0.041
TC (mmol/L)	4.60±1.13	4.48±0.79	4.65±0.95	4.94±0.73	4.51±0.80	4.90±1.14	0.863
HDL –C (mmol/L)	1.28±0.45	1.29±0.55	1.18±0.36	1.13±0.31	1.19±0.30	1.31±0.57	0.762
LDL-C (mmol/L)	2.88±0.93	2.89±1.05	2.64±0.90	2.86±1.22	2.42±0.68	2.74±0.82	0.510

Note:1 mm Hg  = 0.133 kPa; BMI, body mass index; SBP, systolic blood pressure; DBP, diastolic blood pressure; LDL, low-density lipoprotein; HDL, high-density lipoprotein; TG, Triglyceride; TC, Total cholesterol.

### Alcohol consumption and ABI and PAD

As was shown in table 4, we observed a significant increase in ABI with increased alcohol consumption up to daily intake levels of 60 g in men (model 1, *P*<0.001). But the ABI value of individual with daily alcohol level >60 g/d was significantly lower than those never drink or than those with daily alcohol level <60 g/d. After adjusted for the age, ethnicity, body mass index, smoking habits, GLU, and diabetes, the difference remains significant (model 2, *P*<0.001); and when additional confounders including SBP, DBP, TG, TC, HDL-C, and LDL-C were adjusted, this relationship did not change (model 3, *P*<0.001). The frequencies of PAD were different between alcohol categories. Either the unadjusted or multivariate-adjusted odds ratio (OR) for PAD was significantly higher in men who consumed >60.0 g/d [OR  = 3.857, (95% CI: 2.555–5.824); OR = 2.797, (95% CI: 1.106–3.129); OR = 2.878, (95% CI: 1.215–4.018); respectively] and was significantly lower in men who consumed 20.1–40.0 g/d [OR  = 0.330, (95% CI: 0.181–0.599); OR = 0.484, (95% CI: 0.065–0.894); OR = 0.478, (95% CI: 0.243–1.534); respectively] and 40.1–60.0 g/d [OR  = 0.306, (95% CI: 0.096–0.969); OR = 0.267, (95% CI: 0.087–0.886); OR = 0.203, (95% CI: 0.113–0.754); respectively] compared with never drinking, respectively (all *P*<0.01). Neither in unadjusted nor in multivariate-adjusted model the association between ABI and alcohol consumption was significant (all *P*>0.05) in women. Similarly, PAD was not correlated with alcohol intake in women analyzed by each model (all *P*>0.05) (table 5).

**Table 4:** Relation between Alcohol Consumption and ABI or PAD in Men.

	Categories of Alcohol consumption (g/day)						*P* * value
	Never	Former	0.1–20.0	20.1–40.0	40.1–60.0	>60.0	
NO. of subjects	2791	420	1 440	795	214	217	
ABI							
Model 1^a^ ‡	1.078±0.102	1.096±0.104	1.103±0.096	1.095±0.116	1.099±0.094	1.065±0.086	<0.001
Model 2^b^ †	1.073±0.001	1.081±0.005	1.095±0.004	1.081±0.007	1.093±0.008	1.069±0.004	<0.001
Model 3^c^ †	1.074±0.001	1.082±0.005	1.095±0.004	1.077±0.007	1.090±0.008	1.055±0.004	<0.001
PDA (OR)							
Presence of PDA (n, %)	124 (4.44)	26 (6.19)	45 (3.13)	12 (1.51)	3 (1.40)	33 (15.2)	<0.001
Model 1^a^	1	1.419 (0.918–2.195)	0.694 (0.490–0.982)	0.330 (0.181–0.599)	0.306 (0.096–0.969)	3.857 (2.555–5.824)	<0.001
Model 2^b^	1	1.365 (0.923–2.497)	1.086 (0.734–2.758)	0.484 (0.065–0.894)	0.267 (0.087–0.886)	2.797 (1.106–3.129)	0.002
Model 3^c^	1	1.473 (0.094–2.293)	1.121 (0.545–1.767)	0.478 (0.243–1.534)	0.203 (0.113–0.754)	2.878 (1.215–4.018)	<0.001

*P value was calculated by analysis of covariance using all categories of alcohol consumption.

† Mean ± standard error of mean.

‡ Mean ± standard deviation of mean.

a :Unadjusted model;

b :Only adjusted for age, ethnicity, body mass index, smoking habits, GLU, and diabetes.

c :Adjusted for age, ethnicity, body mass index, diabetes, smoking habits, hypertension, SBP, DBP, GLU, TC, HDL cholesterol, and LDL cholesterol;

**Table 5:** Relationship between Alcohol Consumption and ABI and PAD in Women.

	Categories of Alcohol consumption (g/day)						*P* * value
	Never	Former	0.1–5.0	5.1–10.0	10.1–20.0	>20.0	
NO. of subjects	5 147	82	1038	174	271	201	
ABI							
Model 1^a^ ‡	1.098±0.096	1.092±0.095	1.100±0.089	1.086±0.112	1.098±0.082	1.102±0.094	0.338
Model 2^b^ †	1.098±0.002	1.097±0.005	1.105±0.004	1.088±0.008	1.100±0.008	1.104±0.004	0.301
Model 3^c^ †	1.097±0.002	1.095±0.005	1.104±0.004	1.090±0.007	1.102±0.008	1.106±0.004	0.203
PAD (OR)							
Presence of PDA (n, %)	333 (6.47)	6 (7.32)	60 (5.78)	11 (6.32)	14 (5.71)	16 (7.96)	0.802
Model 1^a^	1	1.141 (0.493–2.640)	0.887 (0.668–1.177)	0.976 (0.525–1.815)	0.788 (0.455–1.364)	1.250 (0.741–2.109)	0.804
Model 2^b^	1	1.101 (0.518–2.634)	0.568 (0.158–1.550)	0.875 (0.316–1.402)	0.764 (0.331–1.535)	1.214 (0.716–2.046)	0.199
Model 3^c^	1	1.144 (0.732–2.986)	0.598 (0.337–1.830)	0.886 (0.414–1.517)	0.765 (0.298–1.815)	1.301 (0.898–2.643)	0.187

*P value was calculated by analysis of covariance using all categories of alcohol consumption.

† Mean ± standard error of mean.

‡ Mean ± standard deviation of mean.

a :Unadjusted model;

b : Only adjusted for age, ethnicity, body mass index, smoking habits, GLU, and diabetes.

c : Adjusted for age, ethnicity, body mass index, smoking habits, BP, diabetes, GLU, TC, HDL cholesterol, and LDL cholesterol;

## Discussion

In this study, we observed a significant increase in ABI with increased alcohol consumption up to daily intake levels of 60 g in Chinese men but not in women. And heavier drinking (>60 g/d) can increase the risk of PAD in men but not in women. In other words, our results indicated that in Chinese men, alcohol consumption was associated with peripheral atherosclerosis, and consumption of less than 60 g/d had an inverse association with peripheral atherosclerosis whereas consumption of 60 g/d or more had a positive association.

Several studies have demonstrated that a low ABI is an independent predictor of cardiovascular risk[7], [15]–[19], low ABI is more frequent in patients with cardiovascular risk factors such as smoking, diabetes, and hypertension and is inversely correlated with other measures of vascular disease, including microalbuminuri [20] and carotid intimal-medial thickness [21]–[22]. In the previous studies, much research focus on the relationship between alcohol and carotid IMT, [14], [23]–[28] one of the risk factors of CVD, although they have come to conflicting conclusions. Therefore, the ABI, another risk factor of CVD, its association with alcohol intake should be worth paying close attention to. A few studies have assessed this relation but the results are discrepancy. Mukama et al. observed that consumption of 1–13 alcoholic drinks per week was associated with lower risk of hospitalized lower extremity arterial disease (LEAD) in older adults, with a similar trend for risk of decline in ABI over time, but heavier drinking was not associated with lower risk [29]. Jepson et al. found greater alcohol consumption was related to a higher ABI in males but not in females [30]. And Vliegenthart observed an inverse association between alcohol consumption and PAD in nonsmoking men and women [31]. Fabsitz *et al*. found current alcohol consumption was significantly negatively associated with PAD [32].

In our analysis, we observed moderate drinking may be a protective factor but heavier drinking could be a risk factor for peripheral arteriosclerosis in Chinese men. The mechanisms, which may link alcohol intake to ABI, are largely unknown. Theoretically, the ethanol and nonalcoholic components of alcoholic beverages have possible favorable effect on endothelium by reducing intercellular adhesion molecule-1, vascular cell adhesion molecule-1, and E-selectin expression of vascular endothelium, as well as monocyte adhesion [33]. Alcohol could also enhance nitric oxide synthase expression and subsequent nitric oxide release from endothelial cells, leading to vasoprotective impact [34], [35]. Therefore, alcohol consumption and ABI, like alcohol intake and IMT or other factors for CVD, showed a decreasing risk function at moderate drinking. But in our analysis, this relationship was observed only in men but not in women. This discrepancy may be explained (1) in our study, due to the affection by Chinese traditional culture, the majority of women were never drinkers, those who did drink, had a narrower range, and the alcohol consumption was categorized differently in men and women considering the metabolic difference by sex [14]. This fact may be a possible reason for this discrepancy; (2) alcohol ingestion results in significant alterations in sex hormone levels and function [36]. Alcoholic men and women often display different phenotypic changes due to an inability to maintain appropriate hormone balance. And the sex hormone have been demonstrated a significant association with cardiovascular disease, including arteriosclerosis; [37], [38] (3) differences between men and women in histological characteristics and the stage of peripheral arteriosclerosis under study may be, at least in part, another important factor.

This study has several limitations. First, this study is questionnaire-derived estimates of self-reported alcohol intake which have been criticized because of underreporting, especially by heavy drinkers. Therefore in the present study, we validated self-reported alcohol intake against asking not only themselves but also their family members at the same time face to face or by telephone. Second, in the present study, because of the absence of a real and important confounder-the social disparities in our database, we did not inclusive this variable in the multivariable analysis. This may underestimate or overestimate the real association of alcohol with peripheral arteriosclerosis. Third, in the present study, we did not investigate the role of different type of alcoholic beverages, such as wine, beer, and liquor. Fourth, in our study, the number of women with never drink was far more than other alcohol consumption category, which was the fact of dinking in women of China. This fact may underestimate the association of alcohol intake with peripheral arteriosclerosis.

Several strengths include the inclusion of a large three-ethnic cohort of individuals from the community. We used uniform protocols in the three ethnic groups including questionnaires, anthropometric measurements, assessment of conventional risk factors, and the ABI measure. We separated former drinkers who had stopped drinking for health or other reasons from nondrinkers. Former drinkers have different characteristics from nondrinkers, and analyzing their data separately provided more accurate results.

## Methods

### Ethics Statement

The present study was conducted in accordance with the Declaration of Helsinki guidelines, and informed consent was obtained from each individual according to a protocol approved by the Ethics Committee of the First Affiliated Hospital of Xinjiang Medical University.

### Subjects

The Cardiovascular Risk Survey (CRS) study is a multiple-ethnic, community-based, cross-sectional study designed to investigate the prevalence, incidence, and risk factors for cardiovascular diseases and to determine the genetic and environmental contributions to atherosclerosis, CAD and cerebral infarction (CI) of Chinese Han, Uygur, and Kazakh population in Xinjiang of west China from October 2007 to March 2010. We used a stratified sampling method to select a representative sample of the general population of Chinese Hans, Uygurs, and Kazakhs of this area. Seven cities (Urumqi, Kelamayi, Hetian, Zhaosu, Fukang, Tulufan, and Fuhai) were chosen and, based on the government record of registered residence, one participant was randomly selected from each household. In this way, a total of 14 618 participants (5 757 Hans, 4 767 Uygurs, and 4 094 Kazakhs), were randomly selected from 26 villages of these seven cities and were invited to participate. Patients with a previous cardiovascular event, such as myocardial infarction, stroke, and heart failure, have been excluded (677 of Han, 605 of Uygur, 490 of Kazakh) from the analysis. In addition, those whose data were incomplete (8 of Han, 20 of Uygur, 2 of Kazakh) and individual with ABI>1.3 (26 subjects in total) were excluded. Finally, 12 790 individuals (87.49%) were analyzed in the present study.

### Alcohol consumption

In our study, to assess the drinking status of the study population, we used four questions as follows. The fist question was “ Prior to this study, have you ever drunk alcoholic beverages? ”; The second questions was “In the 12 months before the date of this study, do you drink alcoholic beverages? ”; The third question was “On a day when you do drink alcohol, how many drinks do you usually have?”; The forth question was “How often do you have a drink containing alcohol, per week?”. The persons who answered “no” to both the first question and the second question were classified as never-drinkers. The persons who answered “yes” to the first question and “no” to the second question were classified as former drinkers. The persons who answered “yes” to both the first question and the second question were defined as current drinkers. If the persons were defined as current drinkers, the third and the forth question must be answered. The amount of alcohol consumed per day was calculated from the average number of alcoholic beverages consumed. We categorized daily ethanol intake in grams into six categories for men: former, none, 0.1 to 20.0, 20.1 to 40.0, 40.1 to 60.0, and 60.1 gram or more and for women: never, former, 0.1–5.0, 5.1–10.0, 10.1–20.0, and >20.0 gram or more. Participants were asked whether they changed their usual pattern of consumption and, if so, whether they have increased or decreased their consumption.

### ABI Measurement

The ABI was measured in all subjects using the form ABI/PWV (VP1000; Colin, CO.,Ltd., Komaki, Japan) which is a device with four cuffs that can simultaneously measure blood pressure levels in both arms and both legs and automatically calculate the ankle brachial pressure index (ABI). The measure procedure was described by Ohnishi et al [39]. Briefly, the methods as follows: After a 5-min rest, subjects were evaluated in the supine position. The cuff was inflated to 10 mm Hg above SBP and deflated at 2 mm Hg/s. The first reappearance of the arterial signal was taken as the SBP. To calculate the ABI, the SBP at each ankle site (posterior tibial and dorsalis pedis arteries) was divided by the higher of the two brachial pressures. The lower of the average ABIs from the two legs was used in the analyses [40]. PAD was diagnosed if the ABI was less than the cutoff value of 0.9. [30]

### Covariates

We collected information on each subject's medical history and lifestyle characteristics using standardized questionnaires. Systemic arterial hypertension was defined as a systolic blood pressure of ≥140 mmHg and/or a diastolic blood pressure of ≥90 mmHg [41], on at least two separate occasions, or anti-hypertensive treatment. Hypercholesterolaemia was defined as a documented total cholesterol value ≥240 mg/dl (≥6.2 mmol/L) or current treatment with cholesterol-lowering medication. Diabetes mellitus was defined as the presence of an active treatment with insulin or an oral antidiabetic agent; for patients on dietary treatment, documentation of an abnormal fasting blood glucose, or glucose tolerance test based on the World Health Organization criteria [42] was required for establishing this diagnosis. Smoking status classifications were current smokers, and never-smokers. All participants underwent a standardized physical examination performed by experienced research staff. Anthropometric measurements were conducted in light clothing and without shoes. Height was measured to the nearest 0.1 cm, and weight was measured with a standard scale in the upright position to the nearest 0.1 kg. Body mass index (BMI) was calculated as weight (kg) divided by height squared (m^2^). Waist circumference was measured to the nearest 0.1 cm at the midpoint between the lower border of the rib cage and the upper hip bone (iliac crest) during expiration.

### Biochemical analysis

Serum was separated from the samples within 30 min and stored at –80°C until analysis. We measured the serum concentration of triglyceride, total cholesterol, HDL and LDL cholesterol, fasting glucose and uric acid using equipment for chemical analysis (Dimension AR/AVL Clinical Chemistry System, Newark, NJ) employed by the Clinical Laboratory Department of the First Affiliated Hospital of Xinjiang Medical University as described previously. [43]–[45]

### Statistical analysis

Data analysis was performed using the computer software *Statistical Package for Social Sciences-SPSS* for Windows (version 17.0). Demographic and clinical characteristics of the study population were expressed as the mean ± standard deviation or as a ratio based on the alcohol consumption categories. ABI were presented as the mean ± standard deviation in the unadjusted model and the mean ± standard error of the mean in the multivariable models. Differences in cardiovascular risk factors and in ABI according to alcohol consumption categories were adjusted for gender and tested by analysis of covariance for quantitative variables. Multivariate associations between alcohol consumption categories and ABI and PAD were tested using analysis of covariance and multiple regressions. To test the linear trends of associations between alcohol consumption and ABI, the categories of alcohol consumption were treated as a continuous variable, excluding former drinkers. The odds ratio for PAD was calculated by binary logistic regression analysis.

## References

[1] Doobay AV, Anand SS (2005). Sensitivity and specificity of the ankle brachial index to predict future cardiovascular outcomes: a systematic review.. Arterioscler Thromb Vasc Biol.

[2] Greenland P, Abrams J, Aurigemma GP, Bond MG, Clark LT (2000). Prevention Conference V: beyond secondary prevention: identifying the high-risk patient for primary prevention: noninvasive tests of atherosclerotic burden: Writing Group III.. Circulation.

[3] Smith SC, Greenland P, Grundy SM (2000). AHA Conference Proceedings. Prevention conference V: beyond secondary prevention: identifying the high-risk patient for primary prevention: executive summary. American Heart Association.. Circulation.

[4] Doobay AV, Anand SS (2005). The sensitivity and specificity of the ankle-brachial index to predict future cardiovascular outcomes: a systematic review.. Arterioscler Thromb Vasc Biol.

[5] Newman AB, Tyrrell KS, Kuller LH (1997). Mortality over four years in SHEP participants with a low ankle-arm index.. J Am Geriatr Soc.

[6] Newman AB, Shemanski L, Manolio TA, Cushman M, Mittelmark M (1999). Ankle-arm index as a predictor of CVD and mortality in the Cardiovascular Health Study: the Cardiovascular Health Study Group.. Arterioscler Thromb Vasc Biol.

[7] Migliacci R, Nasorri R, Ricciarini P, Gresele P (2008). Ankle-brachial index measured by palpation for the diagnosis of peripheral arterial disease.. Family Practice.

[8] Wild SH, Byrne CD, Smith FB, LEE A J, Fowkes FGR (2006). Low Ankle-Brachial Pressure Index Predicts Increased Risk of Cardiovascular Disease Independent of the Metabolic Syndrome and Conventional Cardiovascular Risk Factors in the Edinburgh Artery Study.. Diabetes Care.

[9] Laurin D, Masaki KH, White LR, Launer LJ (2007). Ankle-to-Brachial Index and Dementia: The Honolulu-Asia Aging Study.. Circulation.

[10] Fagrell B, De Faire U, Bondy S, Criqui M, Gaziano M (1999). The effects of light to moderate drinking on cardiovascular diseases.. J Intern Med.

[11] Corrao G, Rubbiati L, Bagnardi V, Zambon A, Poikolainen K (2000). Alcohol and coronary heart disease: a meta-analysis.. Addiction.

[12] O'Keefe JH, Bybee KA, Lavie CJ (2007). Alcohol and cardiovascular health: the razor-sharp double-edged sword.. J Am Coll Cardiol.

[13] Di Castelnuovo A, Costanzo S, Bagnardi V, Donati MB, Iacoviello L (2006). Alcohol dosing and total mortality in men and women: an updated meta-analysis of 34 prospective studies.. Arch Intern Med.

[14] Schminke U, Luedemann J, Berger K, Alte D, Mitusch R (2005). Association between alcohol consumption and subclinical carotid atherosclerosis: the Study of Health in Pomerania.. Stroke.

[15] Nunez D, Morillas P, Quiles J, Cordero A, Guindo J (2010). Usefulness of an abnormal ankle-brachial index for detecting multivessel coronary disease in patients with acute coronary syndrome.. Rev Esp Cardiol.

[16] Wild SH, Byrne CD, Smith FB, Lee AJ, Fowkes FGR (2006). Low Ankle-Brachial Pressure Index Predicts Increased Risk of Cardiovascular Disease Independent of the Metabolic Syndrome and Conventional Cardiovascular Risk Factors in the Edinburgh Artery Study.. Diabetes Care.

[17] Conte MS (2008). Buflomedil in Peripheral Arterial Disease Trials and Tribulations.. Circulation.

[18] Laurin D, Masaki KH, White LR, Launer LJ (2007). Ankle-to-Brachial Index and Dementia The Honolulu-Asia Aging Study.. Circulation.

[19] Criqui MH, Langer RD, Fronek A, Feigelson HS, Klauber MR (1992). Mortality over a period of 10 years in patients with peripheral arterial disease.. N Engl J Med.

[20] Zheng Z, Sharrett AR, Chambless LE, Rosamond WD, Nieto FJ (1997). Associations of ankle-brachial index with clinical coronary heart disease, stroke and preclinical carotid and popliteal atherosclerosis: the Atherosclerosis Risk in Communities (ARIC) Study.. Atherosclerosis.

[21] Kobayashi K, Akishita M, Yu W, Hashimoto M, Ohni M (2004). Interrelationship between noninvasive measurements of atherosclerosis: flow-mediated dilation of brachial artery, carotid intima-media thickness and pulse wave velocity.. Atherosclerosis.

[22] Lorenz MW, Markus HS, Bots ML, Rosvall M, Sitzer M (2007). Prediction of clinical cardiovascular events with carotid intima-media thickness: a systematic review and meta-analysis.. Circulation.

[23] Mukamal KJ, Kronmal RA, Mittleman MA, O'Leary DH, Polak JF (2003). Alcohol consumption and carotid atherosclerosis in older adults: the Cardiovascular Health Study.. Arterioscler Thromb Vasc Biol.

[24] Damiani IT, Gagliardi RJ, Scaff M (2004). The influence of ethanol in alcoholic beverages in extracranial carotid arteries atherosclerosis.. Arq Neuropsiquiatr.

[25] Juonala M, Viikari JS, Kähönen M, Laitinen T, Taittonen L (2008). Alcohol consumption is directly associated with carotid intima-media thickness in Finnish young adults The Cardiovascular Risk in Young Finns Study.. Atherosclerosis.

[26] Djoussé L, Myers RH, Province MA, Hunt SC, Eckfeldt JH (2002). Influence of apolipoprotein E, smoking, and alcohol intake on carotid atherosclerosis: National Heart, Lung, and Blood Institute Family Heart Study.. Stroke.

[27] Zureik M, Gariépy J, Courbon D, Dartigues JF, Ritchie K (2004). Alcohol consumption and carotid artery structure in older French adults: the Three-City Study.. Stroke.

[28] Mukamal KJ, Kennedy M, Cushman M, Kuller LH, Newman AB (2008). Alcohol Consumption and Lower Extremity Arterial Disease among Older Adults: The Cardiovascular Health Study.. Am J Epidemiol.

[29] Jepson RG, Fowkes FG, Donnan PT, Housley E (1995). Alcohol intake as a risk factor for peripheral arterial disease in the general population in the Edinburgh Artery Study.. Eur J Epidemiol.

[30] Vliegenthart R, Geleijnse JM, Hofman A, Meijer WT, van Rooij FJA (2002). Alcohol consumption and risk of peripheral arterial disease: The Rotterdam Study.. Am J Epidemiol.

[31] Fabsitz RR, Sidawy AN, Go O, Lee ET, Welty TK (1999). Prevalence of peripheral arterial disease and associated risk factors in American Indians: The Strong Heart Study.. Am J Epidemiol.

[32] Sacanella E, Estruch R (2003). The effect of alcohol consumption on endothelial adhesion molecule expression.. Addict Biol.

[33] Leikert JF, Rathel TR, Wohlfart P, Cheynier V, Vollmar AM (2002). Red wine polyphenols enhance endothelial nitric oxide synthase expression and subsequent nitric oxide release from endothelial cells.. Circulation.

[34] Wallerath T, Poleo D, Li H, Forstermann U (2003). Red wine increases the expression of human endothelial nitric oxide synthase: a mechanism that may contribute to its beneficial cardiovascular effects.. J Am Coll Cardiol.

[35] Kauhanen J, Kaplan GA, Goldberg DE, Salonen R, Salonen JT (1999). Pattern of alcohol drinking and progression of atherosclerosis.. Arterioscler Thromb Vasc Biol.

[36] Eagon PK (2010). Alcoholic liver injury: Influence of gender and hormones.. World J Gastroenterol.

[37] Olsen TS, Andersen KK (2010). Female survival advantage relates to male inferiority rather than female superiority: A hypothesis based on the impact of age and stroke severity on 1-week to 1-year case fatality in 40,155 men and women.. Gend Med.

[38] Traish AM, Kypreos KE (2010). Testosterone and cardiovascular disease: An old idea with modern clinical implications.. Atherosclerosis.

[39] Ohnishi H, Isobe T, Saitoh S, Kikuchi Y, Takagi S (2003). Pulse wave velocity as an indicator of atherosclerosis in impaired fasting glucose: The Tanno and sobetsu study.. Diabetes care.

[40] Ali Z, Sarcia P, Mosley TH, Kullo IJ (2009). Association of Serum Myeloperoxidase with Peripheral Arterial Disease.. Vasc Med;.

[41] Guidelines Subcommittee. (1999). World Health Organization-International Society of Hypertension Guidelines for the Management of Hypertension.. J Hypertens.

[42] World Health Organization Study Group (1985). Diabetes mellitus.. WHO Tech Rep Ser.

[43] Xie X, Ma YT, Fu ZY, Yang YN, Ma X (2009). Association of polymorphisms of PTGS2 and CYP8A1 with myocardial infarction.. Clin Chem Lab Med.

[44] Yang YN, Wang XL, Ma YT, Xie X, Fu ZY (2010). Association of interaction between smoking and CYP 2C19*3 polymorphism with coronary artery disease in a Uighur population.. Clin Appl Thromb Hemost.

[45] Xie X, Ma YT, Fu ZY, Yang YN, Xiang Ma (2009). Haplotype analysis of the CYP8A1 gene associated with myocardial infarction.. Clin Appl Thromb Hemost.

